# Integrated analysis reveals the dysfunction of intercellular communication and metabolic signals in dilated cardiomyopathy

**DOI:** 10.1016/j.heliyon.2024.e26803

**Published:** 2024-02-22

**Authors:** Rui Shi, Xiue Ma, Mi Zhou, Xin Xie, Liang Xu

**Affiliations:** a Department of Obstetrics and Gynecology, East Hospital, Tongji University School of Medicine, Shanghai, China; b Key Laboratory of Arrhythmias of the Ministry of Education of China, Research Center for Translational Medicine, Shanghai East Hospital, Tongji University School of Medicine, Shanghai, 200120, China; c Department of Cardiovascular Surgery, Ruijin Hospital, Shanghai Jiao Tong University School of Medicine, Shanghai 200025, China; d Department of Cardiology, Shanghai East Hospital, Tongji University School of Medicine, Shanghai, 200120, China; e Department of Cardiology, Shanghai General Hospital, Shanghai Jiaotong University School of Medicine, 201620, China

**Keywords:** Cell diversity, Dilated cardiomyopathy, Intercellular communication, Metabolic signaling pathway, Single-nucleus sequencing

## Abstract

**Aims:**

Dilated cardiomyopathy refers to a heart muscle condition characterized by structural and functional irregularities in the myocardium that are not related to ischemia. Due to diverse etiologies such as genetic mutations, infections, and exposure to toxins, dilated cardiomyopathy can lead to substantial morbidity and mortality despite advances in the management of heart failure in dilated cardiomyopathy patients. We sought to analyze the characteristics of cell-cell communication and the metabolic signaling pathways in dilated cardiomyopathy.

**Methods and results:**

The single-nucleus sequencing data of left ventricle samples were acquired from two donor datasets and two dilated cardiomyopathy datasets. Three dilated cardiomyopathy bulk-sequencing datasets were included to determine the shared dilated cardiomyopathy-specific alterations in differentially expressed genes and signaling pathways. Using “CellChat,” we analyzed intercellular communication to grasp how cell clusters interact and to map out the impaired signaling pathways in both donor and dilated cardiomyopathy conditions. Gene set enrichment analysis was applied to compare the metabolic signaling before and after dilated cardiomyopathy. We showcased how cell clusters exhibited abnormal cell-to-cell signaling transduction and how each cell type displayed dysfunctional metabolic signaling pathways through the integration of various datasets. The crucial ligand-receptor signaling contributing to outgoing or incoming signaling of dilated cardiomyopathy was identified in a cell-type dependent way, and the cell-specific metabolic alterations in glucose, lipid and amino acid were determined. The expression of gene pairs in BMP and NOTCH signal, as well as the gene expression in the arginine metabolism was validated.

**Conclusions:**

We reveal the key signals and metabolic pathways for dilated cardiomyopathy adaptation and maintenance, providing potential targets for dilated cardiomyopathy interference.

## Highlights

• In DCM, the communication between cells is abnormal.
• Various cell types exhibit distinct alterations in their cell-cell signaling transduction pathways in DCM.
• Dysfunctional metabolic changes occur in DCM.
• Glucose, lipid, and amino acid pathways are impaired in a cell specific manner in DCM.

## Graphical abstract

A summary figure for DCM pathology. Toxins, viruses, and genetic mutations play pivotal roles in instigating abnormalities within the heart, leading to disruptions in vital processes such as cell-to-cell communication, signal transduction, and metabolism. These disturbances ultimately culminate in the onset of DCM.

**Figure:**
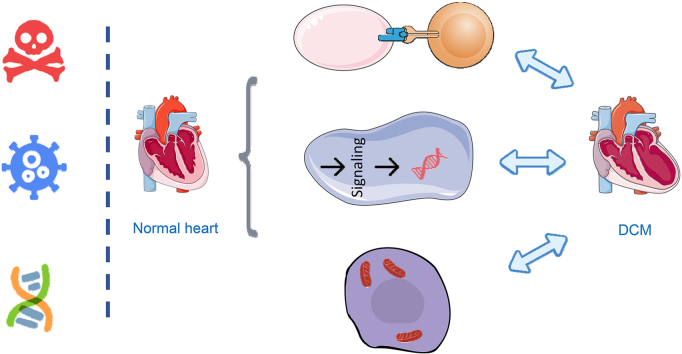

## 1 Introduction

Dilated cardiomyopathy (DCM) is a clinical diagnosis characterized by left ventricular or biventricular dilation and systolic dysfunction in the absence of abnormal loading conditions or coronary artery disease [1]. DCM is one of the most common causes of heart failure and the leading global indication for heart transplantation. The prevalence of DCM is 40 cases per 100,000 individuals. Genetic causes affect 20–35% of cases, and acquired causes of cardiomyopathy include toxins, drugs, infectious agents, and endocrine disturbances [[2], [3], [4]].

Even with progress in patient care, the varied causes and clinical symptoms of DCM continue to pose challenges in achieving a prompt diagnosis. As DCM eventually cause impaired heart contractility, the treatment for DCM aims to prevent or treat heart failure. Defining the probable cause of DCM and identifying aetiology-driven personalized approach will benefit DCM patients. The intricate origins of DCM suggest that combining molecular phenotyping with clinical phenotypes could offer valuable insights into the disease's characteristics and progression. The tissue-level comparisons of mRNA and protein have uncovered disease-specific transcriptional states [5,6]. Recent advances in single-cell and single-nucleus RNA-sequencing (snRNA-seq) technologies put the frontiers of our knowledge at single-cell resolution [7,8].

Taking the advantage of high-throughput sequencing and multi-omics technologies, we acquired massive information pertaining to common and rare cell populations within the healthy or diseased hearts [9,10]. Distinct transcriptional states of multiple cell types were identified and validated, and region-specific transcriptional signatures were observed. However, little is known regarding the interactive functions among human cardiac cell types. Therefore, it is necessary to explore the functional relevance of cell diversity within human cardiac cell populations, and to examine the molecular mechanism of its pathological changes in the disease state.

In this study, we integrated three bulk-seq datasets and four snRNA-seq datasets containing two donor datasets and two DCM datasets to perform a comprehensive analysis for cell diversity, intercellular communication, and metabolic states that promoted disease progression. Our study may provide new biomarkers or targets for DCM prognosis and treatment.

## 2 Methods

snRNA-seq data collection.

The raw count matrices for 14 adult heart samples were sourced from the Human Cell Atlas (https://www.heartcellatlas.org/). Additionally, snRNA-seq raw counts from 7 adult human myocardial samples were acquired via the Broad Institute's Single Cell Portal (https://singlecell.broadinstitute.org/single_cell/study/SCP498/transcriptional-and-cellular-diversity-of-the-human-heart). Furthermore, counts matrices of DCM heart samples, encompassing snRNA-seq data from 25 healthy donors and 13 failing human hearts, were retrieved from the GEO database (GSE183852). The counts matrices of another DCM heart samples were obtained from the Broad Institute's Single Cell Portal (https://singlecell.broadinstitute.org/single_cell/study/SCP1303/single-nuclei-profiling-of-human-dilated-and-hypertrophic-cardiomyopathy), which contained 16 healthy donors and 11 DCM hearts. Summarily we included 61 healthy hearts and 24 DCM hearts. To compare the gene expression across the heart tissue, we only included the samples of left ventricles of hearts from the four datasets, which comprised of 75 healthy samples and 33 DCM samples. The data details were condensed into Table S1, Table S2, and Fig. S1.

### 2.1 Quality control and analysis of snRNA-seq data

We used R package Seurat (4.0.5) [11,12] to process the data from the four datasets. Considering the differential sequencing depth, we used an adaptive threshold for the data quality control. Cells were filtered out based on criteria: those with feature and count numbers beyond the range of median ±3 x median absolute deviation (MAD), and those displaying mitochondrial and ribosomal gene percentages lower than median + 3 x MAD were excluded. And we excluded the cells expressing hemoglobin genes. The 21,801 shared genes across four datasets were used in the integrated data. Doublets were discarded using DoubletFinder (version 1.0.0) [13]. As a result, 461,652 individual nuclei were eligible for further investigation.

### 2.2 Integration of snRNA-seq datasets and cell annoatation

Integration of all samples was performed using the R package Harmony (0.1.0) [14]. Filtered cells were normalized and scaled, and the RunHarmony command was used to generate harmonized dimension reduction components. The clustering process was executed utilizing the Seurat commands FindNeighbors and FindClusters, employing 80 principal components and a resolution set at 0.08. Cell types were annotated using the R package singleR (1.10.0) [15] with the in-house built single-cell references, and verified by canonical marker genes.

### 2.3 Identification of differentially expressed genes (DEGs) in bulk-seq and snRNA-seq data

The raw DCM bulk-seq datasets were obtained by downloading data from the GEO database (GSE116250, GSE126569, and GSE135055). The reads from the donor and DCM samples were processed to remove the adapter sequences by trim_galore (https://github.com/FelixKrueger/TrimGalore/), mapped against the GRCh38.p13.genome using hisat2 [16]. The number of reads in genes was counted by featureCounts [17]. Then, the Deseq2 package [18] was used to determine the fold changes to the control condition. The cutoff of Log 2 fold change value > 1 (2-fold absolute value) and adjusted P-value <0.05 were used for determining significant DEG transcripts.

In the snRNA-seq data, the “findMarkers” function within the Seurat R package conducted differential expression analysis, employing the “Wilcoxon” significance test to compare the target cell type between donor and DCM conditions. These genes were filtered by P < 0.05, pct.1 and pct.2 > 0.2, and |log2FC| > 0.5.

### 2.4 Gene ontology (GO) and Kyoto encyclopedia of genes and genomes (KEGG) analysis

To investigate the function of DEGs, we used the R package clusterProfiler (4.0.5) [19,20]. To showcase shared DEGs across the three bulk-seq datasets, the biological process, cellular component, and molecular function sub-ontologies were illustrated. To visualize the GO annotation across bulk-seq datasets, we adopted “compareCluster” with the function “enrichGO”.

To annotate the function of DEGs in each cell cluster and between donor and DCM conditions, “compareCluster” was utilized with the function “enrichGO” and “enrichKEGG” to delineate the gene ontolog and KEGG pathway for each cell type.

### 2.5 Gene set enrichment analysis (GSEA) analysis in bulk-seq datasets

GSEA was performed by the clusterProfiler R package with the gene set collections from the MSigDB (v7.4 MSigDB). The pathways were sourced from the MSigDB/GSEA resource c2.cp.kegg.v7.4.entrez.gmt, available at (https://www.gsea-msigdb.org/gsea/msigdb/). The GSEA analysis used the whole transcriptomic data in each bulk-seq data with a P-value cut-off as 0.05. The dot plot was made by ggplot 2 R package. (3.3.6)

### 2.6 Cell-cell communication analysis using CellChat

Intercellular communication was deduced from the snRNA-seq data using CellChat (version 1.1.3) [21]. This method allows for the quantitative characterization and comparison of intercellular communications across cell populations, relying on known ligand-receptor interactions. To thoroughly analyze cell-cell communication, we utilized the entire built-in database encompassing “secreted signaling,” “ECM-receptor,” and “Cell-Cell Contact."

### 2.7 Single sample gene set enrichment analysis (ssGSEA)

The ssGSEA was applied to explore the different metabolic pathways in each cluster under donor and DCM conditions. The metabolic pathway gene sets were obtained from previous study [22], which contained the list of metabolic gene sets from KEGG and REACTOME database (https://github.com/wu-yc/scMetabolism/). The sampled subset of the integrated snRNA-seq data was used to compare the enrichment levels of metabolic signatures between donor and DCM conditions. The enrichment of the metabolic signatures for each cell was quantified using the R package “GSVA” (https://www.bioconductor.org/packages/release/bioc/html/GSVA.html) with the method of ssGSEA. The comparison of significance between donor and DCM conditions within each cluster was conducted using the R package “limma” [23].

### 2.8 DCM mouse models

All animals used in the study were maintained in accordance with the Guide for the Care and Use of Laboratory Animals (NIH Publication), and the experimental procedures with animals were approved by the Institutional Animal Care and Use Committee of Tongji University School of Medicine (#2019TJdx49). The αMHC-MerCreMer-Lox system was widely used for targeting the floxed genes of mouse myocardium, in which cardiac specific promoter α-myosin heavy chain (αMHC) ensured the expression of Cre recombinase only within cardiomyocytes, and tamoxifen (an estrogen analog) was added to activate Cre expression. However, Cre recombinase can be cardiotoxic [24]. Evidence has shown that large doses of tamoxifen overstimulate Cre, resulting in decreased cardiac function through enhanced myocyte death/fibrosis and DNA damage [[25], [26], [27]], with the phenotype similar to DCM in human beings. Therefore, we employed mice that expressed inducible αMHC-MerCreMer to validate the gene expression. The investigations were performed using 30 male αMHC-MerCreMer mice aged at 8 weeks. These mice were identified by polymerase chain reaction (PCR) using a 2 x Rapid Taq Master Mix (P222-01, Vazyme, Jiangsu, China). The Cre animals was identified by the primers (forward: TCGATGCAACGAGTGATGAG; and reverse: TCCATGAGTGAACGAACCTG). The Cre mice were injected intraperitoneally with tamoxifen in corn oil (90 mg/kg body weight, 10 mg/ml stock solution, Sigma, St. Louis, MO, USA) once per day for seven consecutive days. The echocardiography was monitored until the mice developed heart failure.

### 2.9 Real-time PCR

Myocardium was homogenized in Trizol (Thermo Fisher Scientific) and reverse-transcribed into cDNA using oligo-dT and random primers (RR037A; Takara Bio, Kawasaki, Japan). The gene expression was quantified by real-time quantitative PCR using SYBR Green (QPK-201, TOYOBO, Japan) as the reporter dye. The expression level of the target mRNA was normalized to the expression of the internal reference gene β-actin and calculated using the method of the 2 − (ΔΔCt) method. The primers used in this study were listed in Table S3.

### 2.10 Western blot analysis

Proteins were extracted using RIPA buffer (Beyotime, Zhejiang, China) including protease inhibitor cocktail tablets (Roche Applied Science, Mannheim, Germany). Subsequently, the extracted proteins were separated by SDS-PAGE (Invitrogen), and transferred to PVDF membranes. The membranes were then incubated with the following primary antibodies: Ryr2 (ab302716, Abcam), Atp2a2 (#9580, Cell signaling technology, USA), Pln (MA3-922, Invitrogen), Notch3 (55114-1-AP, Proteintech), Phospho-Smad2/3 (#8828, CST), Phospho-Stat3 (#9145, CST), Stat3 (sc-8019, Santa cruz), and β-Actin (sc-47778, Santa cruz). Following washing, the blots were treated with IRDye 800 conjugated secondary antibodies (072‐07‐15‐06 or 072‐07‐18‐06, LI-COR Biosciences, Lincoln, NE, USA) at room temperature for 1 h. Images were captured using the Odyssey infrared imaging system and analyzed with the Odyssey Application Software v2 (LI-COR Biosciences).

### 2.11 Statistical analysis

GraphPad Prism 8 (GraphPad Software, San Diego, CA) was used for the statistical analysis of the data with unpaired *t*-test, or one-way ANOVA analysis. P values lower than 0.05 were considered significant.

## 3 Results

### 3.1 Integration of snRNA-seq data from healthy and DCM samples revealed the cellular complexity in heart tissues

To characterize the cellular composition of both healthy and DCM hearts, we obtained four snRNA-seq datasets comprising 85 hearts (61 healthy and 24 DCM patients). This allowed us to outline the cellular diversity present in these heart tissues. Following rigorous quality control measures, we retained a total of 461,652 cells derived from 75 healthy samples and 33 DCM samples for subsequent analysis (Fig. 1a and Fig. S2). Using Harmony method, we integrated 108 samples from four projects and two conditions. We defined the main cell types using the SingleR [15] and canonical cell marker genes. Consequently, the integrated dataset revealed 12 distinct clusters (Fig. 1b). These cells were well-integrated in projects (Fig. S3), and disease conditions (Fig. 1c). Cell identities were further validated by cell-specific marker genes (Fig. 1d, and Table S4). Consistent with previous report [15], cardiac myocytes and fibroblasts were the most abundant cell types in heart tissues, occupying more than 40% of the total cells. Endothelial cells and pericytes account for 30% of the sum, and the number of myeloid cells comprises of 10% of the total. The rest cell types, smooth muscle cells (SMCs), endocardial cells, lymphoid cells, mast cells, neurons, lymphatic endothelial cells and adipocytes make up 10% of the cell population.

**Fig. 1:**
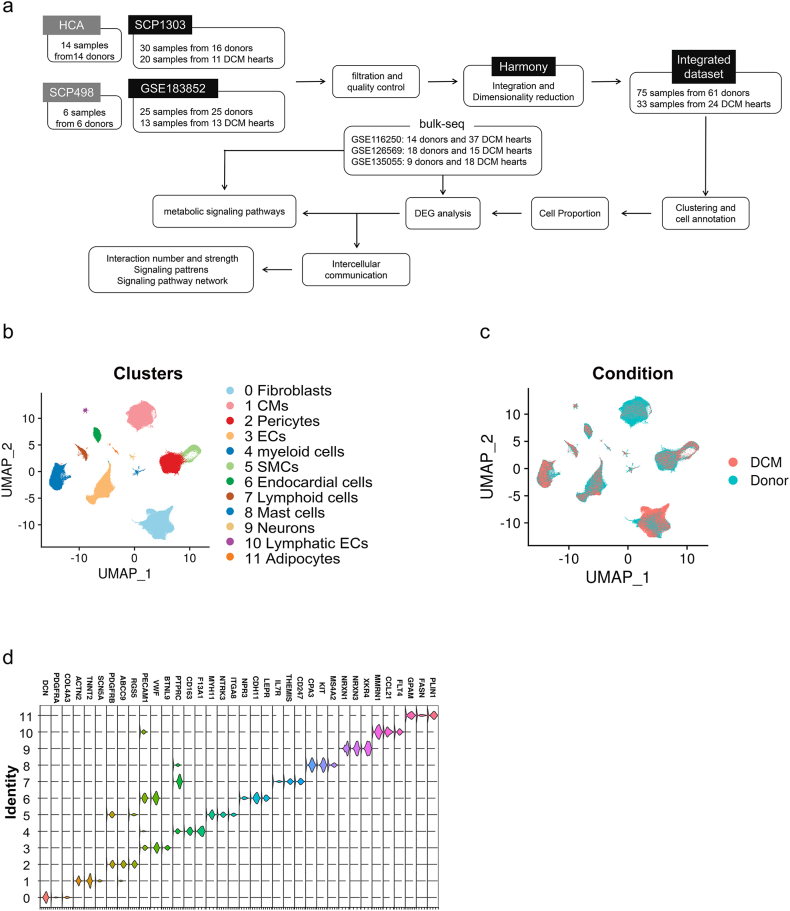
**Integration of public snRNA-seq datasets of human donor and failing hearts.** (a) Workflow of snRNA-seq data processing, quality control, dimensionality reduction, clustering and analyzing. (b) UMAP visualization of 461,652 cells and 12 clusters. (c) The integrated effects of DCM conditions. (d) Violin plot depicting the expression of canonical marker genes in each cluster.

### 3.2 Disease-specific alteration in cell populations of DCM

Considering the diversity of cells within heart tissues, our subsequent focus was on exploring changes in cell proportions within DCM. Illustrated in Fig. 2ab, the sample composition was illustrated by cell clusters and grouped by projects. We further examined the difference of cell populations between donor and DCM samples in each cluster. As expected, the cardiac myocytes (cluster 1) were dramatically decreased and the fibroblasts (cluster 0) were significantly increased in DCM samples (Fig. 2c). Compared with the donor hearts, DCM hearts exhibited statistically increased in endothelial cells (cluster 3), myeloid cells (cluster 4), SMCs (cluster 5), lymphoid cells (cluster 7), neurons (cluster 9), and lymphatic endothelial cells (cluster 10), and remarkable decrease in pericytes (cluster 2). Interestingly the cell proportion of endocardial cells (cluster 6), mast cells (cluster 8), and adipocytes (cluster 11) were not significantly changed in DCM samples. We next adopted the GO (Fig. 2d) and KEGG (Fig. 2e) annotation for these cell clusters to reveal the cell-type specific regulation in the disease state. For example, the increased proportion of fibroblasts may be associated with extracellular matrix structural constituent, ECM-receptor interaction and PI3K-Akt signaling pathway. The lymphoid cells played the active roles in T/B cell receptor signaling transduction and regulation of GTPase regulator activity and protein serine/threonine kinase activity. Taken together, the differential cell ratio may be involved in the progress of DCM.

**Fig. 2:**
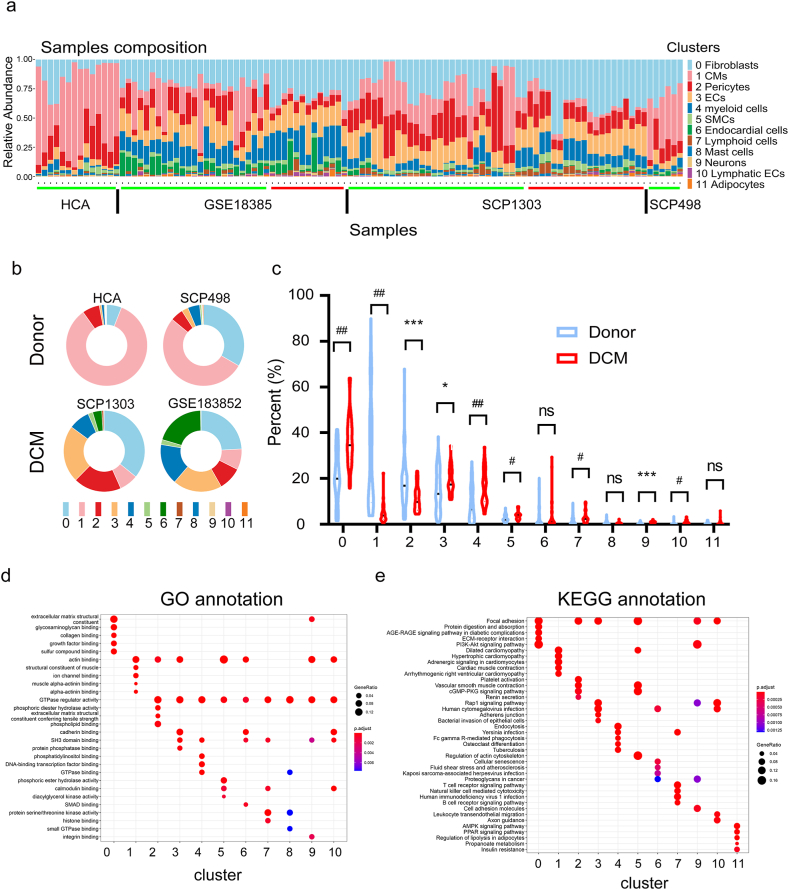
**Cell component in the donor and DCM conditions.** (a) The cell composition of each cluster in each sample. The green lines indicated the nonfailing samples, and the red lines indicated the DCM samples. (b) The cell composition of each cluster in each project. (c) The percentage of donor and DCM cells in each cluster. *, P < 0.05; **, P < 0.01; ***, P < 0.001; #, P < 0.0001; ##, P < 0.00001; ns, no significance. The functional comparisons for the DEGs annotated by GO annotation (d) and KEGG annotation (e) for each cluster.

### 3.3 Identification of dysfunctional signaling pathways in a cell-type dependent manner in DCM

To reveal the DEGs in heart tissues of DCM samples, we analyzed the gene expression from donor and DCM conditions in each cluster. A collective of 1372 genes exhibited differential expression (Table S5). These genes were illustrated in Fig. 3a with the top5 up-regulated and down-regulated DEGs in each cluster labeled. We further compared the signaling pathways enriched in each cluster (Fig. 3b). The “ubiquitin meditated proteolysis” was involved in all clusters, suggesting a universal protein degradation change. The “lysosome” occurred in myeloid cells, indicating the function of macrophages. The AMPK signaling pathway played a role in cardiac myocytes and adipocytes, implying the metabolic dysfunction in DCM. Intriguingly cellular senescence was found in lymphoid cells, suggesting an aged immune system in DCM.

**Fig. 3:**
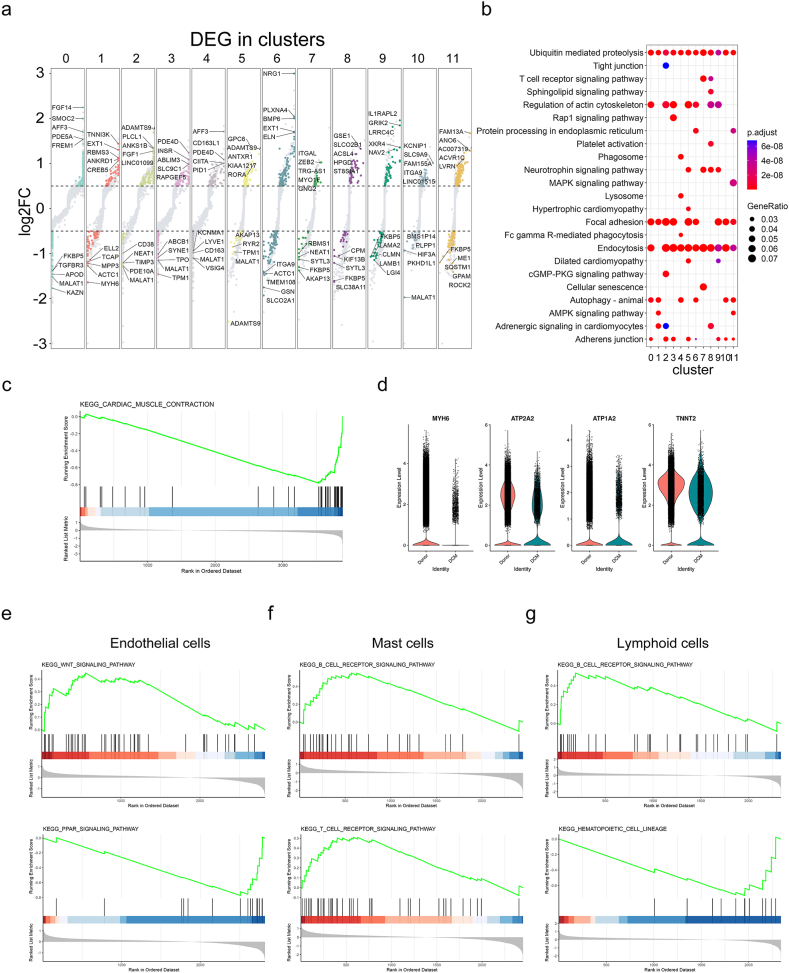
**The DEGs in clusters and under the DCM condition.** (a) Dot plot of the DEGs between donor and DCM conditions in each cluster. The DEGs were colored with the cutoff of |log2FC| > 0.5, the P value < 0.05, and pct.1 and pct.2 > 0.2. The top5 up-regulated and down-regulated DEGs were labeled. (b) The comparison of GO annotation for the DEGs between donor and DCM conditions in each cluster. (c) The contraction of cardiac muscle in cardiac myocytes was down-regulated revealed by GSEA analysis and the gene expression analysis (d). The GSEA enrichment profile in endothelial cells (e), mast cells (f) and lymphoid cells (g).

In addition to the DEG analysis, we performed gene set enrichment analysis, which provided a functional analysis of snRNA-seq data at the level of gene sets. As expected, we found the cardiac myocytes in DCM hearts to be negatively enriched in cardiac muscle contraction (Fig. 3c). Accordingly the contraction-associated genes such as MYH6, ATP2A2, ATP1A2, and TNNT2 were down-regulated (Fig. 3d). In endothelial cells, GSEA revealed the activation of WNT signaling and inhibition of PPAR signaling pathway in DCM (Fig. 3e). Although T/B cell receptor signaling pathways were boosted in mast cells (Fig. 3f) and lymphoid cells (Fig. 3g), the hematopoietic cell linage was attenuated in lymphoid cells. These results indicate that disease-specific and cell-type specific pathway alteration was induced in DCM.

### 3.4 Dysfunction of cell-cell communication in DCM

Next we attempted to investigate the cell-cell communication between donor and DCM hearts. We used “CellChat” to construct the communication network between donor and DCM samples to characterize the alterations in signaling pathways. Our results demonstrated that the global number and strength of interactions were both reduced (Fig. 4a), and each cluster had differential number and strength of interactions (Fig. 4b). The number of interactions in myeloid cells (cluster 4), SMCs (cluster 5) and endocardial cells (cluster 6) were generally decreased, and the strength of fibroblasts (cluster 0), cardiac myocytes(cluster 1), and lymphatic endothelial cells (cluster 10) were universally increased. Notably the impairment of one signaling may induce the enhancement of another signaling. In endocardial cells, the incoming pathway such as COLLAGEN signaling pathway was diminished from fibroblasts (Fig. 4c), but the outgoing pathway such as BMP signaling pathway was enhanced to a few cell types such as fibroblasts and cardiomyocytes (Fig. 4d). This phenomenon occurred even in the same signaling pathway. In NOTCH signaling pathway, the destruction of communication between SMCs and pericytes/lymphoid cells aroused the projection from SMCs to adipocytes (Fig. 4e). Collectively, the incoming and outgoing signaling patterns between donor and DCM conditions were remarkably changed (Fig. S4).

**Fig. 4:**
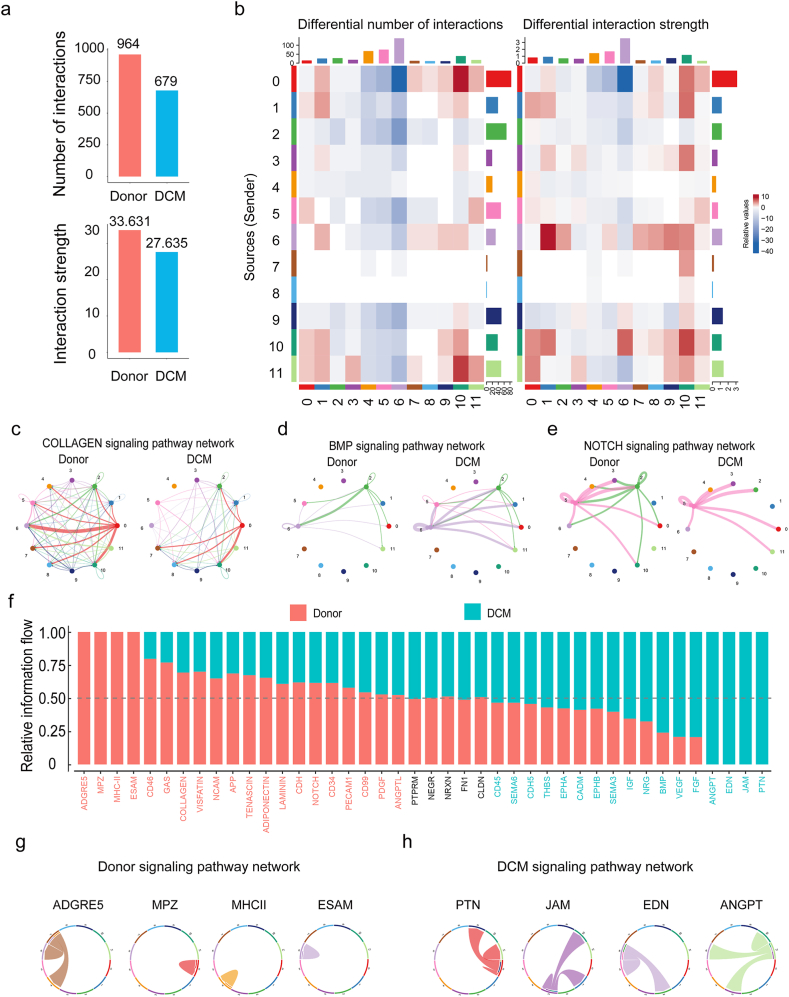
**Cell-cell communication inference in donor and DCM samples.** (a) Bar plot displaying the interaction number and strength between donor and DCM hearts. (b) Heatmap depicting differential numbers of interactions and interaction strength in each cluster. (d) The modes for signaling changing represented by COLLAGEN (c), BMP (d), and NOTCH (e) signaling pathway network. (f) The bar graph showing the overall information flow of each signaling pathway under donor and DCM conditions. Chord diagram showing donor-specific signaling pathways (g) and DCM-specific signaling pathways (h).

To analyze the changes in signaling pathways within DCM, we compared the overall information flow of each pathway. This was characterized by the cumulative communication probability across all pairs of cell groups within the inferred network [21]. Some signaling pathways such as the PTPRM, NEGR, NRXN, FN1, and CLDN were conserved and almost unchanged (Fig. 4f), whereas the COLLAGEN, VISFATIN, NCAM, NRG, BMP, and FGF were dynamically changed. Notably the ADGRE5, MPZ, MHCⅡ, and ESAM were persistently activated in the donor condition, but were shut off in DCM (Fig. 4g), whereas the PTN, JAM, EDN, and ANGPT were turned on in DCM compared with the donor condition (Fig. 4h). These results manifested the general destruction of the cell-cell interaction network in DCM.

### 3.5 Bulk-seq revealed the impairment of metabolic pathways in DCM

To reveal the common signals affected in DCM, we introduced three bulk-seq datasets. DEGs showing up-regulation and down-regulation in each dataset were chosen using a cutoff criterion of P < 0.05 and |log2FC| > 1 (Fig. S5). The shared genes among the three datasets were used for further analysis (Fig. 5a). We discovered that these DEGs were linked to biological processes such as extracellular structure organization, leukocyte aggregation, and SMAD protein signaling transduction. In terms of cellular components, they were associated with the collagen-containing extracellular matrix and the endocytic vesicle lumen. Additionally, in molecular functions, they were related to receptor-ligand activity, signaling receptor activator activity, and cytokine activity (Fig. 5b). We also compared the GO annotation of the DEGs in individual datasets, and found the G protein-coupled peptide receptor activity, gated channel activity, and growth factor activity overlapped (Fig. 5c). We intensively compared the enriched pathways in each dateset by GSEA. Interestingly, while the TGF-β signaling pathway, ECM-receptor interatction were elevated, the metabolism-associated pathways such as glycolysis gluconeogenesis, pyruvate metabolism, and fatty acid metabolism were declined, indicating the metabolism reprogramming in DCM (Fig. 5d). In fact, the significant metabolic heterogeneity of heart tissues was observed in the integrated snRNA-seq dataset (Fig. 5e).

**Fig. 5:**
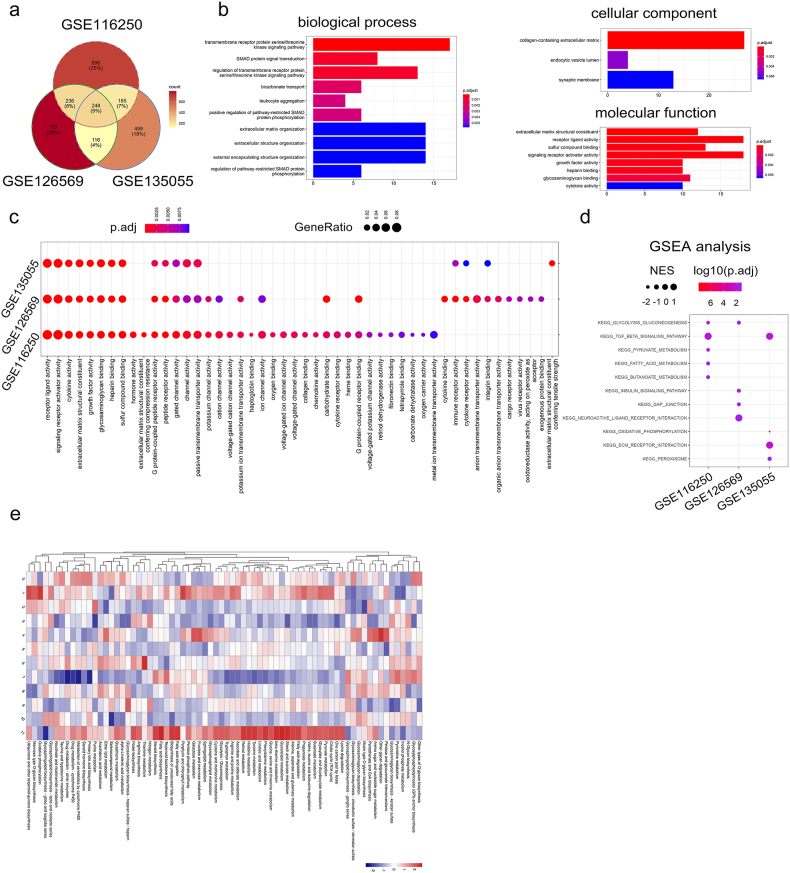
**Functional analysis for the bulk-seq data in DCM.** (a) Venn diagram of the shared and unique DEGs among indicated DCM bulk-seq datasets. (b) The annotations of biological process, cellular component and molecular function for the shared DEGs in the bulk-seq datasets. (c) The GO annotation for the DEGs in three DCM bulk-seq datasets. (d) GSEA analysis for the enriched signaling pathway across three DCM bulk-seq datasets. (e) The metabolic pathway profiles in each cluster.

### 3.6 Abnormal metabolic pathways of cell clusters in DCM

Due to the cell diversity in heart tissues, we next attributed the metabolic abnormality revealed by bulk-seq to the cell type. We computed the score of all 72 active metabolic pathways in each cell cluster under both donor and DCM conditions. Strikingly, the oxidative phosphorylation, glycolysis/gluconeogenesis, and pyruvate metabolism were down-regulated in almost all cell types. Fat metabolism-linked pathways, including fatty acid biosynthesis, and fatty acid elongation were declined prominently in adipocytes (Fig. 6a). This occurred in cardiac myocytes similarly. Interestingly, the butanoate metabolism was decreased in fibroblasts and cardiac myocytes, but increased in endocardial cells, suggesting the enhancement of carbohydrate metabolism in DCM condition. We proceeded to compare the distinct metabolic pathways across all cell types (Fig. 6b). Most cell types share similar metabolic alterations, such as citrate cycle (except cluster 10), and fructose and mannose metabolism. Some cell types have their special features. For instance, pericytes, endocardial cells, and adipocytes developed the change of glycosylphosphatidylinositol (GPI)-anchor biosynthesis. The dysfunction of glycosaminoglycan biosynthesis-keratan sulfate was shown in fibroblasts, endocardial cells, and lymphoid cells. The pantothenate and CoA biosynthesis was observed in mast cells. We next explored the differential metabolic pathways in each cluster under donor and DCM conditions (Table S6). The arginine and proline metabolism and propanoate metabolism were significantly decreased in cardiac myocytes (Fig. 6c). When the biosynthesis of unsaturated fatty aids was decreased, the tryptophan metabolism was elevated in adipocytes (Fig. 6d). These results suggest that the alteration of metabolic signaling was cell type-dependent under the DCM.

**Fig. 6:**
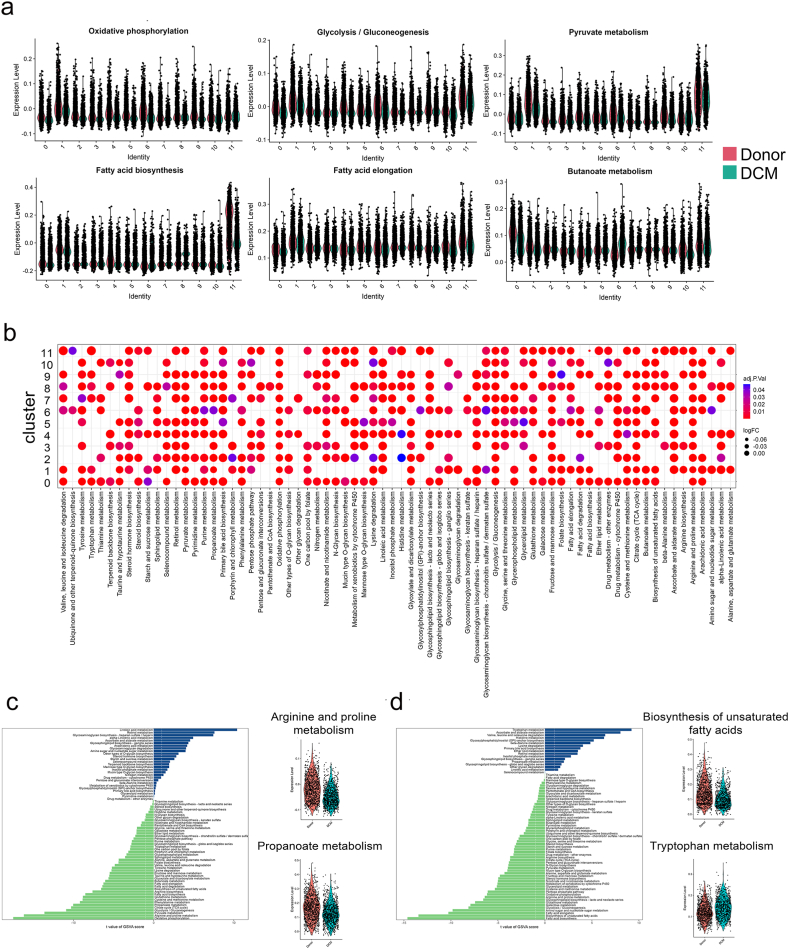
**The metabolic alteration of each cell cluster under the DCM condition.** (a) Violin plot showing the differential metabolic pathways revealed by bulk-seq for DCM. (b) Dot plot showing the comparison of differential metabolic pathways with the metabolic gene sets from the KEGG database. *Left*, bar plot showing the up-regulated and down-regulated metabolic pathways in cardiac myocytes (c), and adipocytes (d); *right*, violin plot showing the representative metabolic pathways in cardiac myocytes (c), and adipocytes (d).

### 3.7 Validation of differentially expressed genes in DCM

To validate the gene expression in dysfunctional signals and metabolic pathways, we collected heart tissues from DCM mouse models and wild type mice. The DCM mice were performed by injection of tamoxifen intraperitoneally and confirmed by echocardiography examination and molecular markers (Fig. 7a–c). The gene expression of signaling gene pairs and metabolic pathways were checked by PCR assay. For easy validation, we chose genes that differentially expressed or dynamically changed in specific cell types. We scrutinized the expression of signaling genes at the individual cell level, subsequently verifying these findings through mRNA detection. As shown in Fig. 7de, the BMP pathway was significantly increased in endocardial cells (cluster 6), and the pathway genes, Bmp 6, Bmpr2, Gdf7, and Bmpr1a were significantly elevated in heart tissues of DCM. By contrast, the pair genes between pericytes (cluster 2), and endocardial cells (cluster 6) and lymphatic endothelial cells (cluster 10), were both decreased (Fig. 7fg). Similarly, the genes, Ckm, Ckmt2, Maob, Sat 1, and P4ha1 in the arginine metabolism were downregulated in the heart tissue of DCM (Fig. 7hi). We further examined the protein expression of genes related to cardiac muscle contraction, cell interaction, and signaling transduction. As seen in Fig. 7j, the calcium handling genes Ryr2, Atg2a2, and Pln were dramatically decreased (Fig. 7j). Moreover, while Notch3 was decreased, the protein expression of Phospho-Smad2, and Phospho-Stat3 was significantly increased (Fig. 7k), indicating the attenuated cell-cell communication in Notch signals and enhanced Tgf-β signals. Taken together, our results suggest that the gene expression in intercellular signals and metabolic pathways are abnormally altered in DCM samples.

**Fig. 7:**
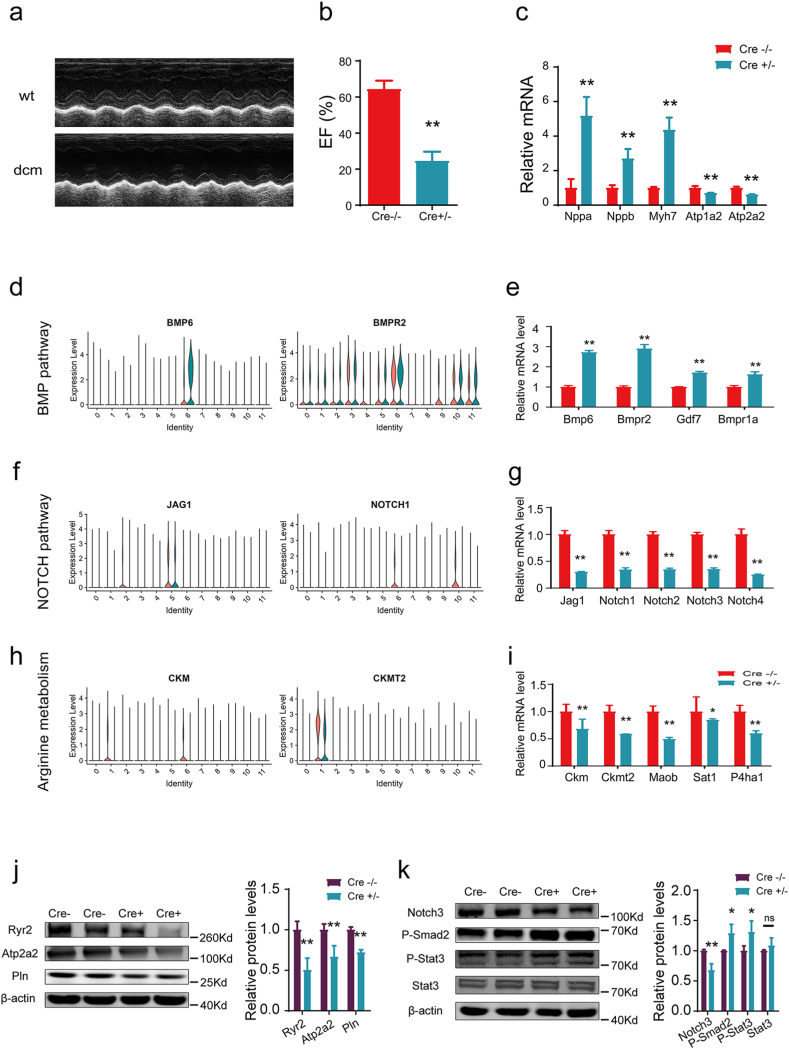
**The validation of gene expression in DCM models.** (a) M-mode echocardiography showing significantly enlarged left ventricle in DCM. (b) The ejection fraction of DCM mice was remarkably decreased after intraperitoneal injection of tamoxifen. EF, ejection fraction. (c) The mRNA levels of the marker and pathway genes Anp, Bnp, Myh7, Atp1a2, and Atp2a2 detected by real-time PCR. (d) Representative gene pair in BMP pathway. (e) qPCR detection of gene pairs in BMP signaling pathway in DCM. (f) Ligand-receptor genes in NOTCH pathway at the single cell level. (g) The mRNA levels of genes in NOTCH pathway under DCM condition. (h) Violin plot showing the gene expression in arginine metabolic pathways. (i) Bar plot showing the downregulated arginine metabolic pathways in DCM heart tissues. Data in c, e, g and i are from three independent experiments, and shown as mean ± s.d. *, P < 0.05; **, P < 0.01. (j) Western blotting analysis for genes associated with cardiac muscle contraction in WT and Cre + DCM mouse models. (k) Western blotting examination for the genes in cell-cell interaction and signaling transduction in the heart of WT and DCM mouse models. Results in j-k are pooled from at least three experiments and shown as mean ± s.d.

## 4 Discussion

In this study, we leverage high-throughput transcriptome sequencing technology to merge four snRNA-seq datasets. This integration aims to uncover the disruptions in signaling pathways and changes in metabolic states within heart tissue between donor and DCM samples. We included more than 450,000 single cells from 108 samples (75 healthy and 33 DCM samples). These cells were grouped into 12 clusters, which indicated the high diversity in the heart tissue. We did not find DCM-associated cell population, but the cell proportion of each cell type was significantly changed in DCM. Consistent with previous report [28,29], the decrease of cardiac myocytes and the increase of fibroblasts were observed in our data. The growth of endothelial cells, SMCs, and lymphatic endothelial cells was paralleled with the reduction of pericytes, indicating the adaption of cell clusters in DCM and the transition from pericyte progenitor-like cells to vascular cells. Cells of the immune system, including myeloid and lymphoid cells, exhibited expansion, indicating the essential role of the immune system in the progression of DCM.

To understand the cell-cell communication in DCM, we used “CellChat” to construct cell crosstalk network [30,31]. We found that compared with the donor condition, there was a notable decrease in both the total number and intensity of interactions in DCM. Various signaling pathways, such as the ADGRE5, MPZ, MHCⅡ, and ESAM were destructed, and replaced by the PTN, JAM, EDN, and ANGPT that were not activated in the donor condition. The cell clusters displayed distinct changes in incoming and outgoing signaling patterns specific to their cell types. For example, the incoming signaling pathways of the LAMININ and COLLAGEN were destroyed in myeloid cells, SMCs, and endocardial cells, but the THBS, and VEGF were strengthened in adipocytes in the outgoing signaling (Fig. S4ab). Even in the single pathway display several modes of cell-cell interactions. As shown in Fig. 4cd, the COLLAGEN and BMP respectively represent the diminished and boosted patterns of signaling transduction, whereas the FGF demonstrated the impairment between SMCs and cluster 0, 1, and 11, and the enhancement between cardiac myocytes and cluster 0, 1, and 11 (Fig. S4c).

In this study, we also included three bulk RNA-seq data in DCM to examine the key regulators in DCM progress. The 248 DEGs were obtained by overlapping these datasets and analyzed by functional annotations, showing the activation of fibrobalsts and the mobilization of immune systems. We further compared the enriched pathways among three datasets. The GSEA revealed the up-regulation of TGF-β signaling pathway and the down-regulation of glucose and lipid metabolism. Alterations in cardiac energy metabolism contribute to the severity of heart failure [32]. Given the differential metabolic states of cell types in heart tissues, we dissected the discrepancies between donor and DCM conditions in each cell type with help of snRNA-seq. We sampled the snRNA-seq data to obtain 23,655 cells and reserve the same cell clusters as the original integrated data. We used previously reported metabolism-related gene signatures [22] to calculate the ssGSEA score for every cell. As expected, the level of oxidative phosphorylation was decreased in all cell types, and the fatty acid metabolism was decreased in adipocytes. Interestingly, we found that endocardial cells was sensitive to the DCM condition. These cells switched the pyruvate metabolism to butanoate metabolism. The increase of pantothenate and CoA biosynthesis was observed in mast cells. Pantothenate, also called vitamin B5, is the precursor for the synthesis of an essential cofactor, CoA, In the metabolic gene signature from REACTOME, we found that the vitamin D calciferol metabolism was unique in endothelial cells and endocardial cells, and the peroxisomal lipid metabolism was specific in pericytes and adipocytes (Fig. S6). These pathways would be served as novel predictive biomarkers associated with DCM development and progression [33,34].

The genetic background contributes to up to 35% of cases of idiopathic disease. Mutations in various genes such as TTN, LMNA, MYH7, BAG3, TNNT2, FLNC, RBM20, SCN5A, PLN, TNNC1, TNNI3, and TPM1 are linked to the development of DCM [3].We investigated the expression of these genes between donor and DCM samples at the single cell level, but no significant differences were observed (Fig. S7). Due to the limitation of 10X Genomics scRNA-seq for detection of SNP, we could not reveal the genetic features across the datasets. In the future, Smart-seq could potentially be implemented to detect mutations at the individual cell level. Furthermore, As tamoxifen-induced DCM model may not represent the more chronic nature and later-onset nature of DCM, we examined the results from doxorubicin-induced DCM mouse models (GSE233644) [35], which was a cardiotoxicity-induced DCM model, representing a more chronic pathological process. The analysis of bulk-seq results showed the similar results with our finding that the signals and cell-cell communication were significantly altered in the DCM hearts. (Fig. S8).

Several limitations still exist in this study. A potential limitation is the source of the DCM samples. Although 24 DCM hearts were included, they were from only two projects. Increasing the number of samples from diverse sources would yield more valuable evidence, capturing a comprehensive range of specific traits among DCM patients. DCM is a heterogeneous condition and the comparative animal models are employed in the DCM research, including αMHC-MerCreMer/doxorubicin-induced DCM models, genetic models with mutations, and virus-induced models. While mouse models provide valuable insights, one should be careful that there models don't fully replicate the complexity of DCM in humans, and species differences may exist. Therefore, findings from mouse models need to be validated in clinical studies. Another constraint pertains to the validation of differentially expressed genes. We only examined the gene expression in cell-cell communication and metabolic signaling pathways at the transcriptional level. The detailed gene expression, including protein expression and distribution may provide more information for the role of these genes in DCM and give clues for understanding the molecular and functional basis. Further studies, including proteomics and metabolomics can reveal molecular details for the occurrence and development of DCM.

To strengthen the quality and depth of the research of DCM, some measures should be taken: 1) Including DCM samples from various origins, which can provide more valuable evidence and represent the comprehensive spectrum of traits in DCM patients. 2) Validation in clinical studies. Given that our research involves mouse models, species differences may exist, so it's crucial to validate our findings in clinical studies to confirm their relevance to human DCM. 3) Incorporating proteomics and metabolomics. Go beyond examining gene expression at the transcriptional level, and consider incorporating proteomics and metabolomics in the next study. These approaches can reveal more molecular details related to the occurrence and development of DCM, enhancing our understanding of the condition.

## 5 Conclusions

In conclusion, we conducted a comprehensive analysis combining snRNA-seq and bulk datasets of DCM, unveiling the intricate cellular diversity within heart tissues. This exposed disruptions in cell-cell communication and metabolic signaling pathways in DCM. Certain distinct metabolic signals might hold promise as new potential biomarkers for DCM prognosis and could offer additional avenues for refining current treatment strategies.

## Funding

This work was supported by the grant from the 10.13039/501100001809National Natural Science Foundation of China for the General Program (82270397 to Liang Xu), and Top-level Clinical Discipline Project of Shanghai Pudong District (PWYgf 2021–01).

## Ethics statement

The experimental procedures with animals were approved by the Institutional Animal Care and Use Committee of Tongji University School of Medicine (#2019TJdx49).

## Data availability statement

The snRNA-seq data in this study were available from the public database: Heart Cell Atlas (https://www.heartcellatlas.org/); Single Cell Portal: SCP498 and SCP1303 (https://singlecell.broadinstitute.org/single_cell); and Gene Expression Omnibus: GSE183852 (https://www.ncbi.nlm.nih.gov/geo/). The bulk-seq data were available from Gene Expression Omnibus: GSE116250, GSE126569, and GSE135055.

## CRediT authorship contribution statement

**Rui Shi:** Writing – review & editing, Writing – original draft, Validation, Methodology, Investigation. **Xiue Ma:** Validation, Methodology. **Mi Zhou:** Visualization, Validation, Methodology, Formal analysis. **Xin Xie:** Writing – review & editing, Resources, Investigation. **Liang Xu:** Writing – review & editing, Validation, Project administration, Funding acquisition, Data curation, Conceptualization.

## Declaration of competing interest

The authors declare that they have no known competing financial interests or personal relationships that could have appeared to influence the work reported in this paper.

## Figures and Tables

**figs1:**
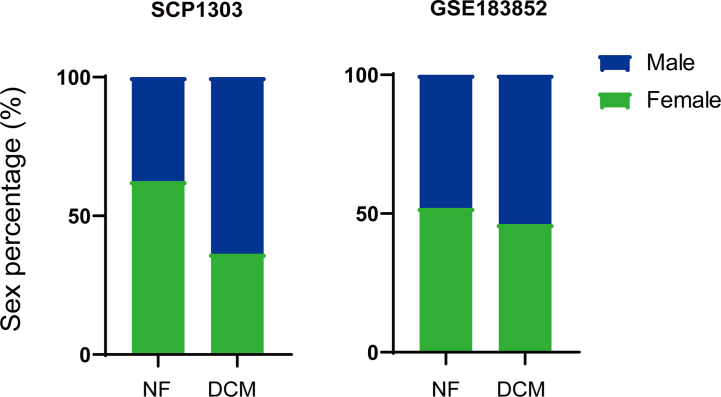


**figs2:**
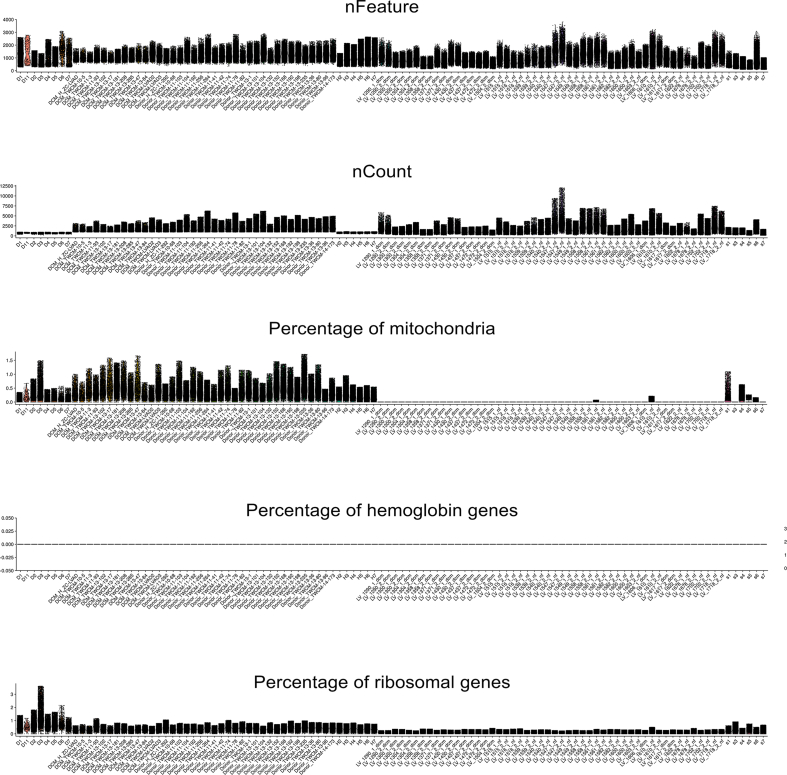


**figs3:**
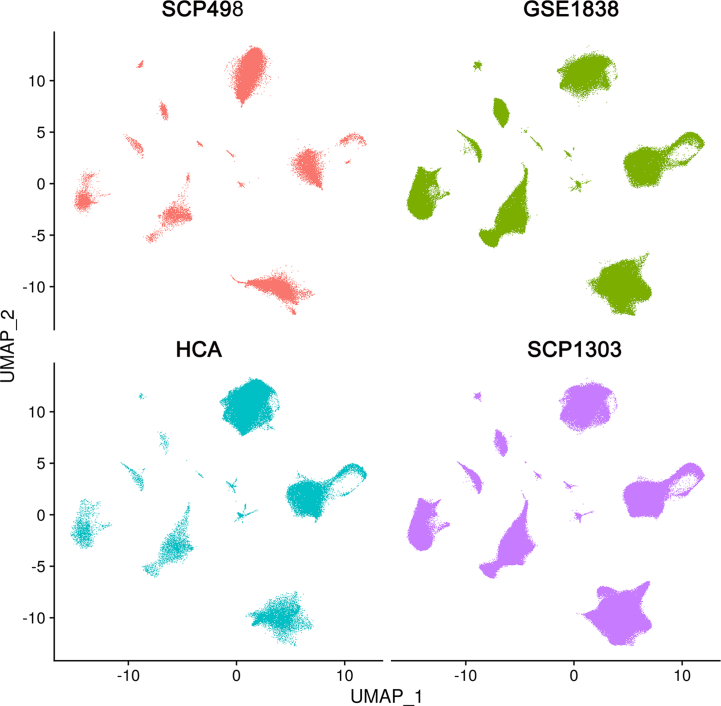


**figs4:**
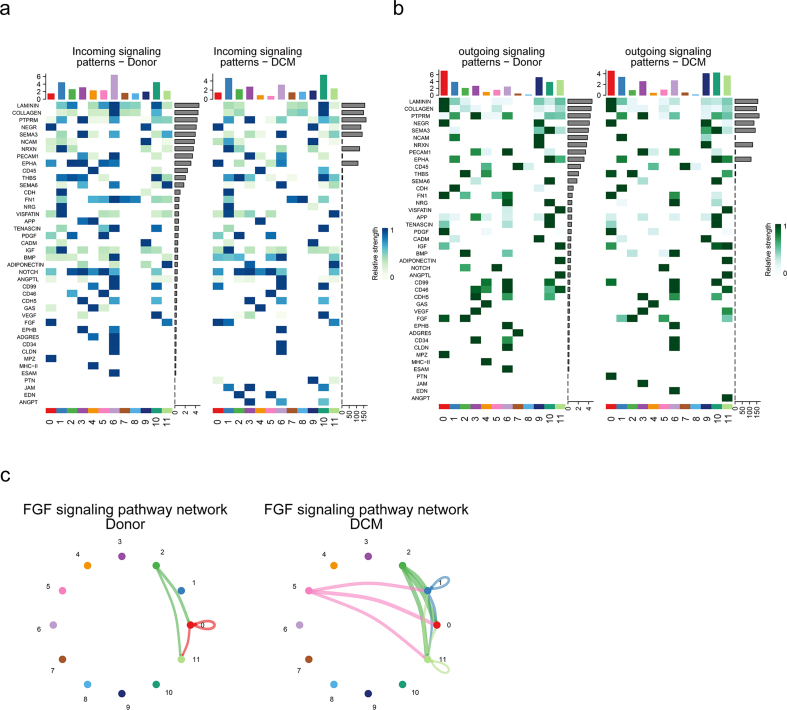


**figs5:**
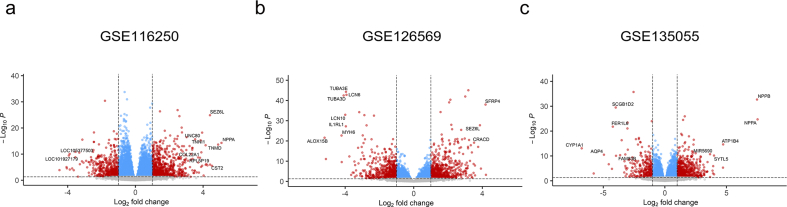


**figs6:**
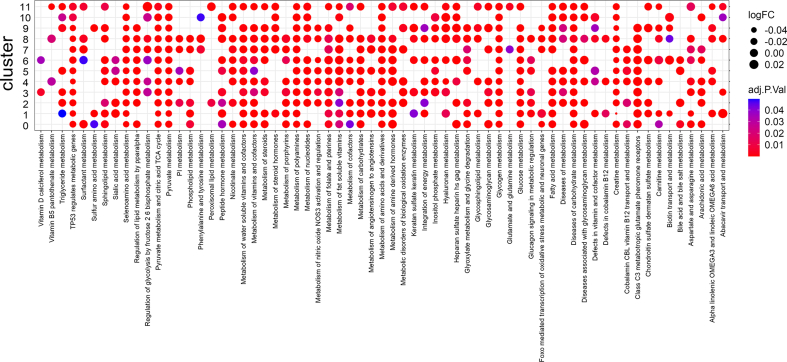


**figs7:**
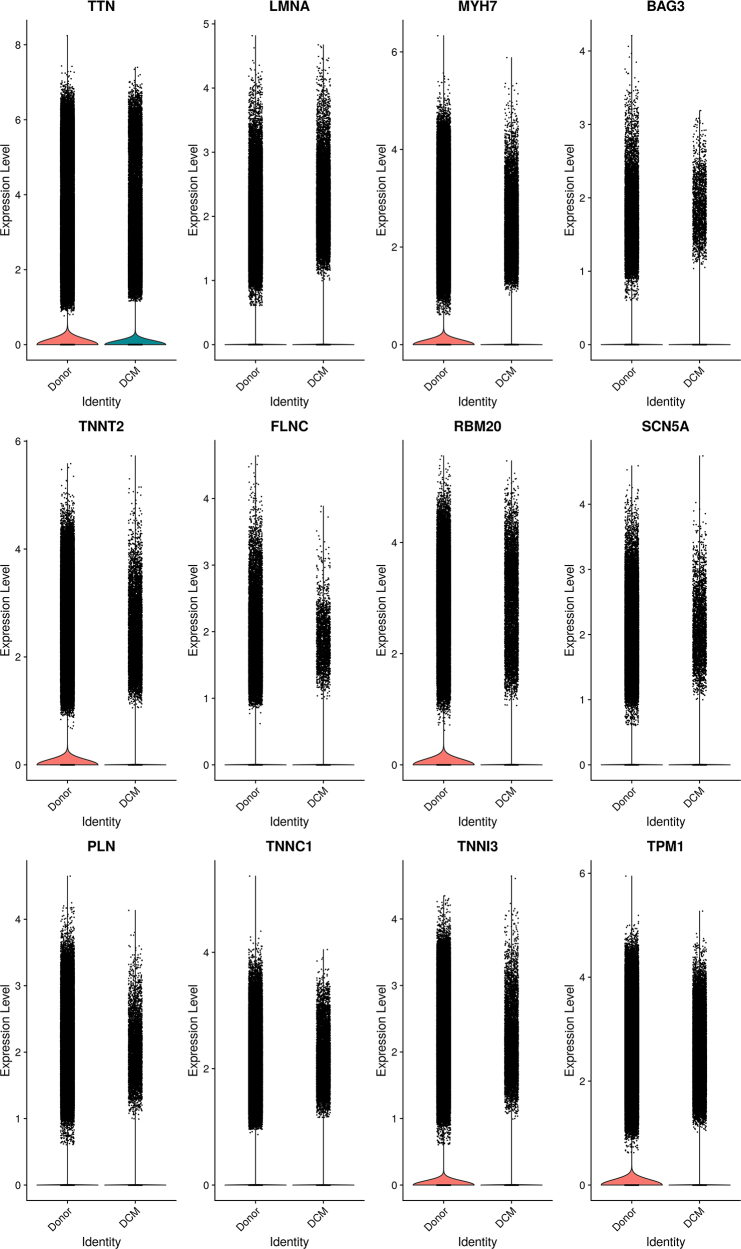


**figs8:**
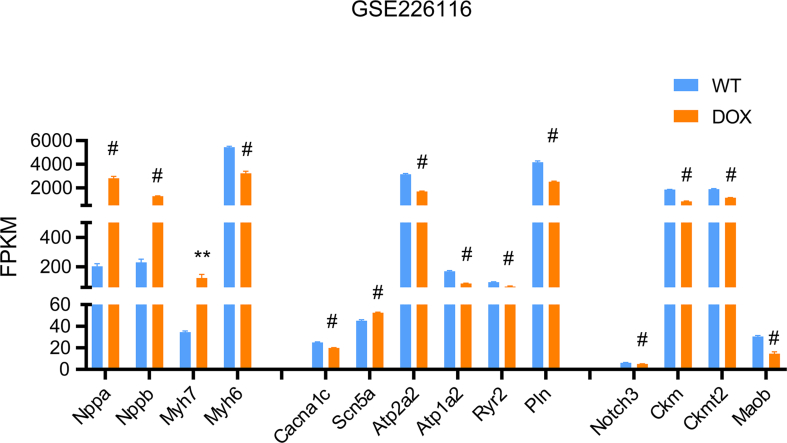


## Abbreviations

Dilated cardiomyopathy: (DCM)
single-nucleus RNA-sequencing: (snRNA-seq)
median absolute deviation: (MAD)
differentially expressed genes: (DEGs)
Gene ontology: (GO)
Kyoto encyclopedia of genes and genomes: (KEGG)
Gene set enrichment analysis: (GSEA)
Single sample gene set enrichment analysis: (ssGSEA)
α-myosin heavy chain: (αMHC)
polymerase chain reaction: (PCR)
smooth muscle cells: (SMCs)
glycosylphosphatidylinositol: (GPI)
