# Supplementary figures and images for: Integrated analysis reveals the dysfunction of intercellular communication and metabolic signals in dilated cardiomyopathy
Source: Heliyon. 2024 Feb 22;10(5):e26803. doi: 10.1016/j.heliyon.2024.e26803 (PMC10907783; doi:10.1016/j.heliyon.2024.e26803)

related to Figure 7j

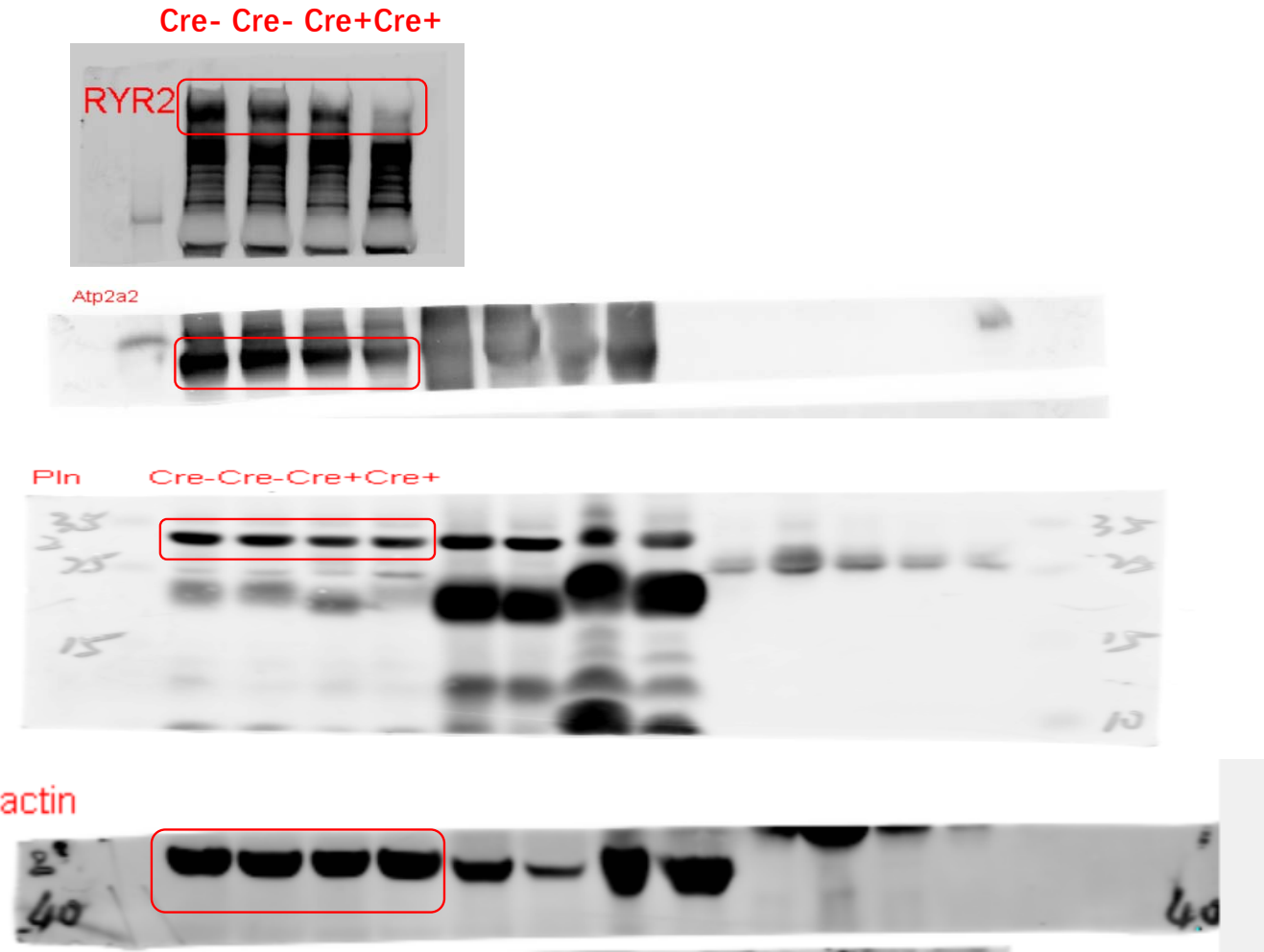

related to Figure 7k

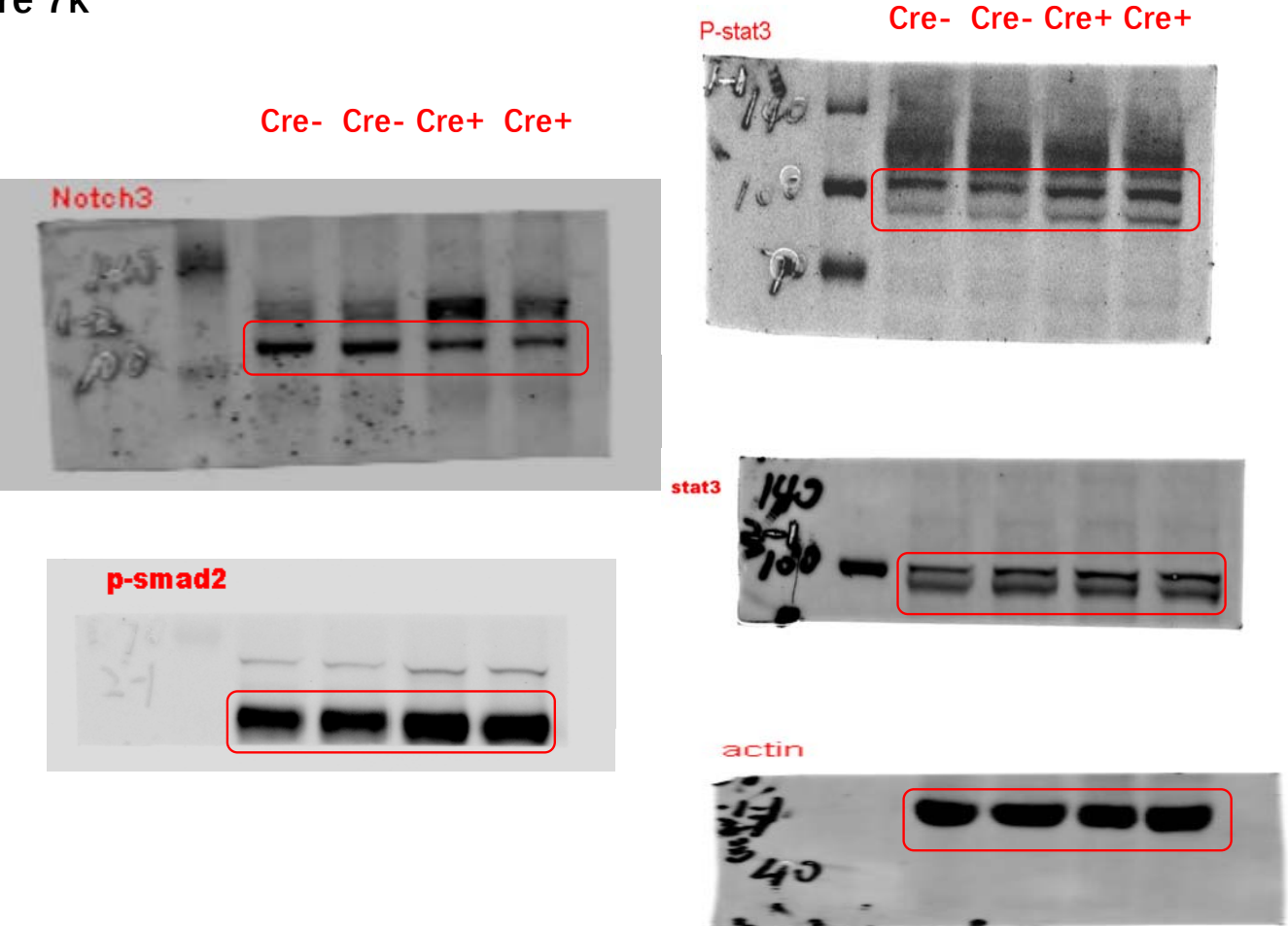

Supplement: Multimedia component 7 [file mmc7.pdf]
